# Determining the appropriate number of particles on a filter to allow small microplastics to be analyzed by microscopy

**DOI:** 10.1016/j.mex.2022.101646

**Published:** 2022-02-24

**Authors:** Haodong Xu, Hisayuki Arakawa

**Affiliations:** Tokyo University of Marine Science and Technology, Konan 4-5-7, Minato-Ku, Tokyo 108-8477, Japan

**Keywords:** Isolation ratio, Micro Fourier-transform infrared spectroscopy, Microplastic, Numerical experiment, Filter, Appropriate quantity, Particle size distribution, Simulator

## Abstract

The concentrations of small microplastics (diameter < 350 µm) on filters are usually determined by microscopy or micro Fourier-transform infrared spectroscopy. The presence of too many particles on a filter will cause the measured number of particles and particle sizes to be incorrect because of overlapping particles. In this study, the appropriate quantity of particles on a filter to allow effective particle analysis to be performed was determined by performing numerical experiments. The larger the number of particles and the larger the particles, the smaller the proportion of isolated particles. An isolation ratio of 99% requires a particle density on the filter of 1.28 particles mm^−2^ if the particle size is 50 µm or of 0.351 particles mm^−2^ if the particle size is 100 µm.

• Appropriate number of particles for small plastic particle analysis was determined.
• Numerical experiments to determine particle distributions on a filter were performed.
• Particle number for a 99% isolation ratio was determined.

## Highlights

• Appropriate number of particles for small plastic particle analysis was determined.
• Numerical experiments to determine particle distributions on a filter were performed.
• Particle number for a 99% isolation ratio was determined.

## Graphical abstract

**Figure:**
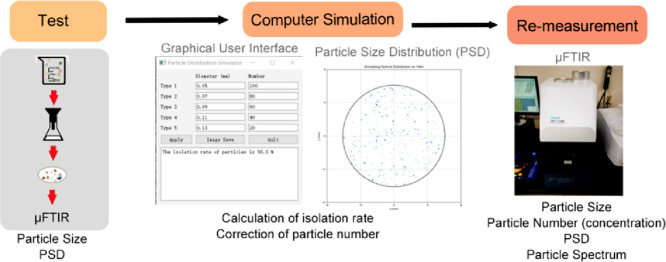

Specifications table

**Table:** 

Subject Area:	*Environmental Science*
More specific subject area:	*Detection and analysis of small microplastics*
Method name:	*Method for determining the appropriate number of particles to allow small microplastics to be analyzed by microscopy*
Name and reference of original method:	*Sample transfer from sieve to microscope, Prume, A.J., Corka, F., Loder, M.G.J., 2021. From sieve to microscope: An efficient technique for sample transfer in the process of microplastics’ quantification. MethodsX, 8, 101,341.*10.1016/j.mex.2021.101341
Resource availability:	*This method contributes to reducing the possibility of erroneous for microscopic measurement and managing the quality of data.*

## Background

In recent years, the concentrations of microplastics in seawater have been determined in many areas around the world [1], [2], [3]. Most of the recent studies on marine microplastics have been conducted using a sampling net with a mesh opening of approximately 350 µm, with the microplastic being confirmed visually and then collected for analysis. As a result, microplastics smaller than 350 µm have not been analyzed very often. However, markedly higher concentrations of these small microplastics (SMPs) with diameters < 350 µm have been found in oceans than those with diameters > 350 µm [4,5]. Because these SMPs appear to have a large negative effect on biological organisms, a detailed understanding of their distribution in the sea area is urgently needed [6,7].

**Fig. 1:**
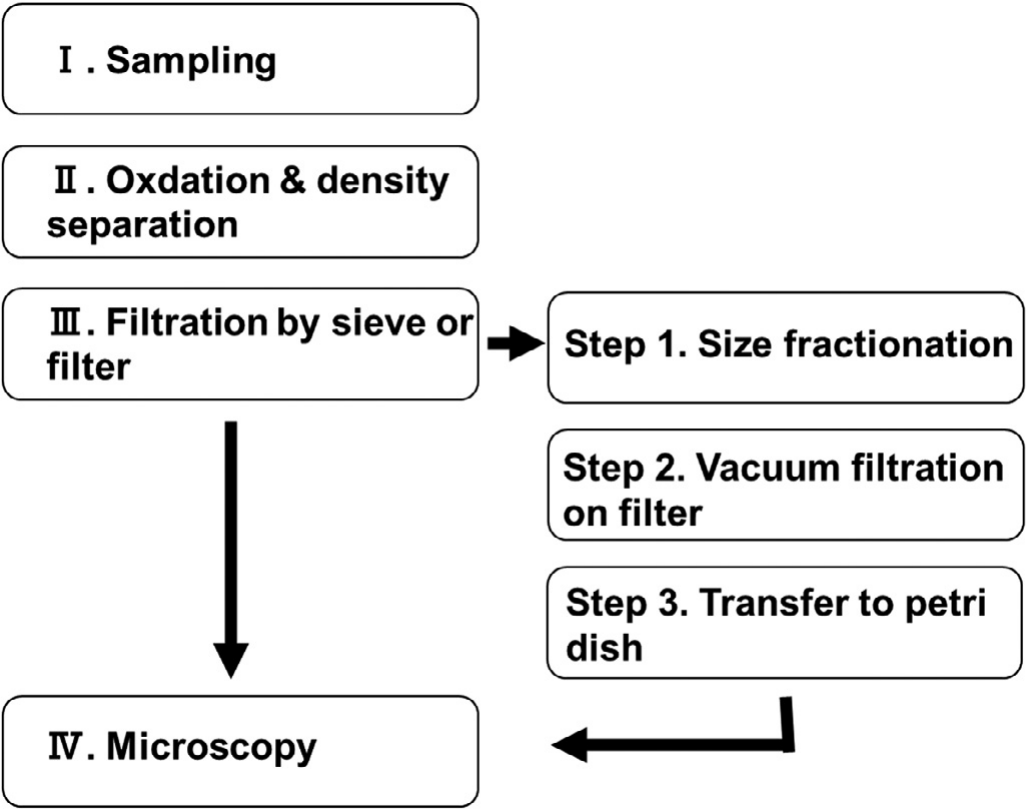
General procedure for analyzing small microplastics from sieving to microscopy. Treatments Ⅰ–IV are the general procedure [12]. Treatments 1–3 are steps between sieving and microscopy [8].

A method that has generally been used to analyze SMPs is shown in Fig. 1. The general sample analysis procedure is shown on the left of Fig. 1, and a procedure developed by Prume et al [8]. is shown on the right.

The collection of SMPs from ocean water (I) is performed using either a pump [9,10] or a net [4,11]. The SMPs are subjected to an oxidation treatment and a density separation process (II) [12] and then collected on a sieve or filter (III). The polymer types of SMPs are analyzed by microscopic Fourier-transform infrared spectroscopy or microscopic Raman spectroscopy (IV). Prume et al [8]. attempted to improve the sample processing techniques between sieving (III) and microscopy (IV). The additional processes between III and IV are size fractionation (Step 1), vacuum filtration (Step 2), and transfer to a petri dish (Step 3). Prior to microscopic analysis, the particles must be collected on a filter (Step 2). If too many particles are collected, the particle sizes and the number of particles may be recorded incorrectly because the particles may come in contact with one another or overlap. If too few particles are collected, the correct particle size distribution cannot be determined.

In this study, the number of particles on a filter required to obtain correct results by microscopic Fourier-transform infrared spectroscopy and microscopic Raman spectroscopy was determined by performing numerical experiments.

## Procedure

In Steps 1 and 2 of the method described by Plume et al [8]., ensuring that an appropriate number of particles become attached to the filter requires determining the particle concentration in the sample and then subjecting an appropriate amount of the sample to vacuum filtration (Step 2). We generated filters with various numbers of particles of different sizes and determined the number of particles that overlapped in each sample.

The program was created using Python version 3.7.3 software (Python Software Foundation, DE, USA). The program is cited in the Supplementary materials.

Each conceptual filter was circular with a diameter of 12.2 mm. The filter structure and pore size affect microplastic quantification [13]. Specifically, a filter with a double layer hole structure with 20 µm pores is recommended for use [14]. However, neither the filter structure nor pore size were specified in this simulation, i.e., we assumed that all the particles of all sizes were retained.

Each particle was a circle with an aspect ratio of 1. A random function was used to generate the particles on a filter. All particles were randomly distributed on the filter and none were beyond the filter edges. The position of each particle was determined using a Cartesian coordinate grid system, with the x and y coordinates independently randomly created. The origin of the Cartesian coordinate grid system was the center of the filter. All coordinates were processed using a precision of eight decimal places.

In the numerical experiments, the loop number of trials, particle size, and number of particles were used to calculate the isolation ratio for the particles. The loop numbers used in the experiments were 10, 50, 100, 500, 1000, 5000, 10,000, and 20,000. The particle sizes were 50, 100, and 150 µm. The numbers of particles were 50, 100, 200, 300, 400, 500, 600, 700, 800, 900, and 1000.

An isolated particle was defined as a particle that was neither overlapping nor tangentially in contact with any other particle. The isolation ratio (%) was calculated using the equation:

(1) $$Isolation\;Ratio=\frac{Isolated\;Particles}{All\;Particles}\times 100$$

**Fig. 2:**
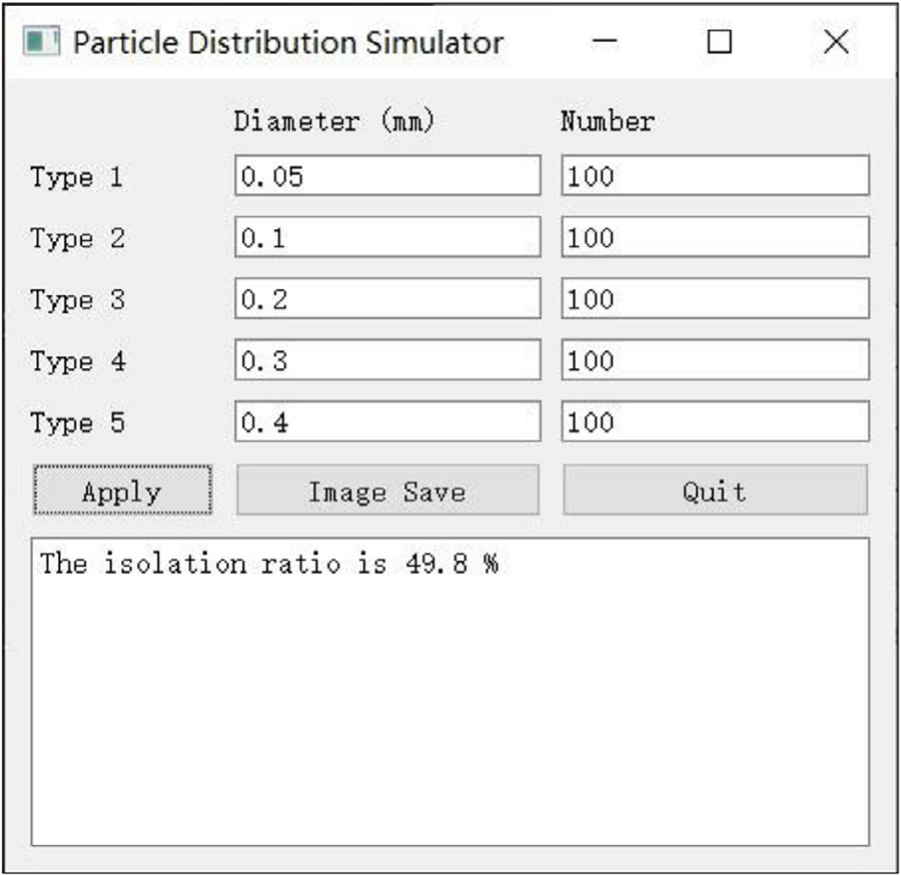
Graphical user interface display, entitled ‘particle distribution simulator’. The input rules are well designed. Only digits are acceptable in the diameter column. An error message will be displayed if a string or unrecognizable symbols are input. Only an integral is accepted in the number column. The ‘Apply’ button runs the program. The ‘Image Save’ button saves the image. The ‘Quit’ button closes the window and application. The isolation ratio will be displayed in the bottom text box.

An interface was created to allow the isolation ratio to be calculated. A concise graphical user interface was built using Python 3.7.3 software to input the particle number and particle size (Fig. 2). Specifically, the graphical user interface toolkit PyQt5-Qt (5.15.2), data analysis package NumPy (1.20.3), and plotting library Matplotlib (3.1.0) were the three main packages that were used to perform the graphical user interface functions. The program allowed five sizes and numbers of particles to be set.

## Method validation

**Fig. 3:**
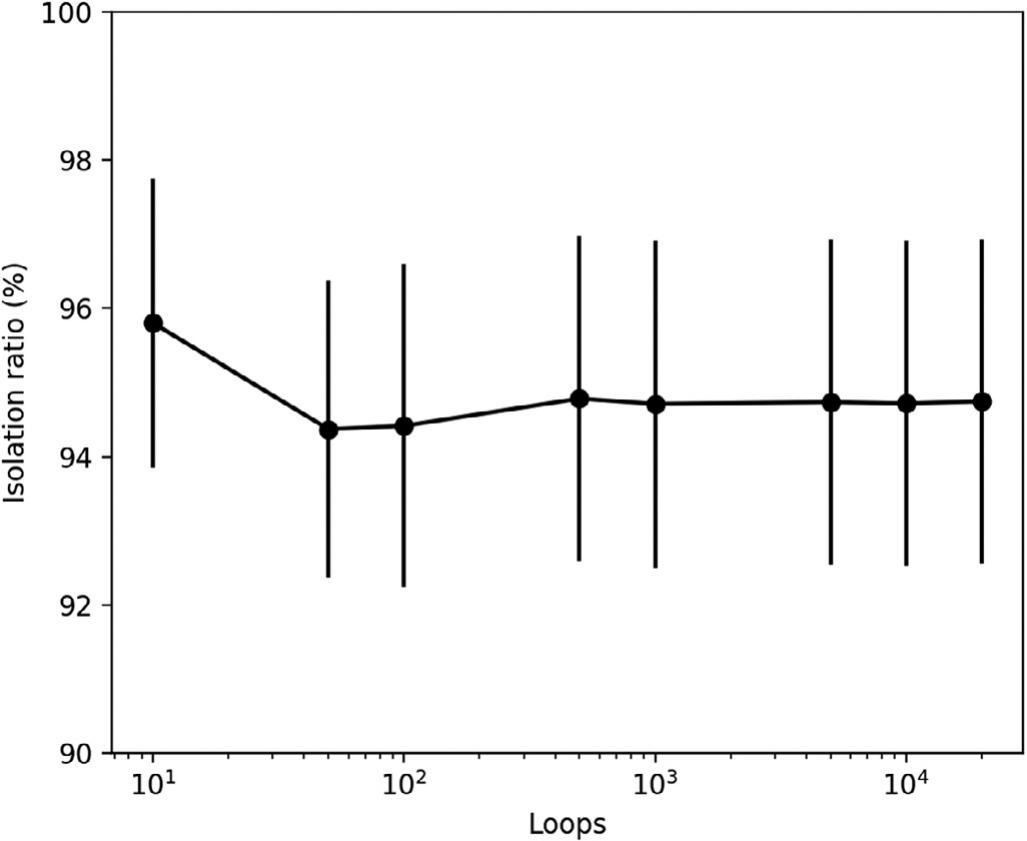
Relationship between the results and loops. Each point is the mean value, and the bar shows the standard deviation. The x-axis is on a logarithmic scale. The results for 10, 50, 100, 500, 1000, 5000, 10,000, and 20,000 loops were compared.

The loop number of trials of numerical experiments was examined first. The isolation ratio and standard deviation for each loop number when 200 particles with a diameter of 100 µm were generated on the filter are shown in Fig. 3. The isolation ratio was large at 10 trial loops, lower at 50–100 loops, and stable at ≥500 loops. Therefore, 500 trial loops were subsequently performed for all the conditions.

**Fig. 4:**
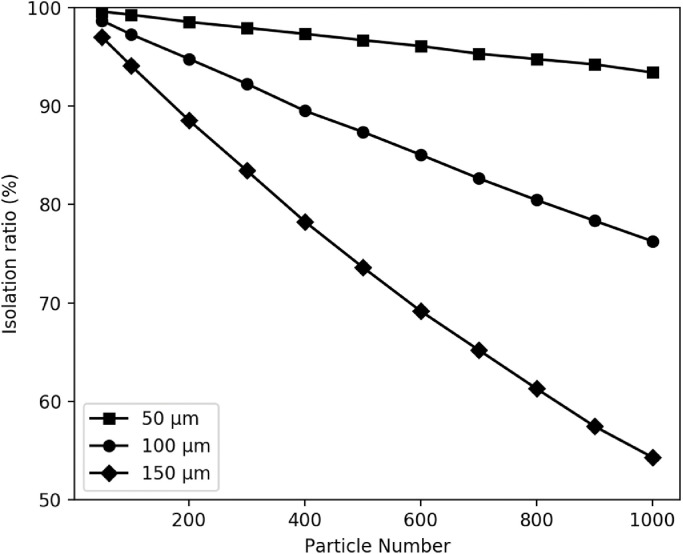
Relationship between the particle number and isolation ratio. The squares are for 50 µm particles, the circles are for 100 µm particles, and the rhombi are for 150 µm particles. Particle numbers 50, 100, 200, 300, 400, 500, 600, 700, 800, 900, and 1000 were used to calculate the mean isolation ratios. In all situations, 500 loops were used.

The relationship between the number of particles and the isolation ratio is shown in Fig. 4. At particle sizes of 50, 100, and 150 µm, the isolation ratio decreased as the number of particles increased. The isolation ratio decreased more dramatically as the particle size increased.

**Fig. 5:**
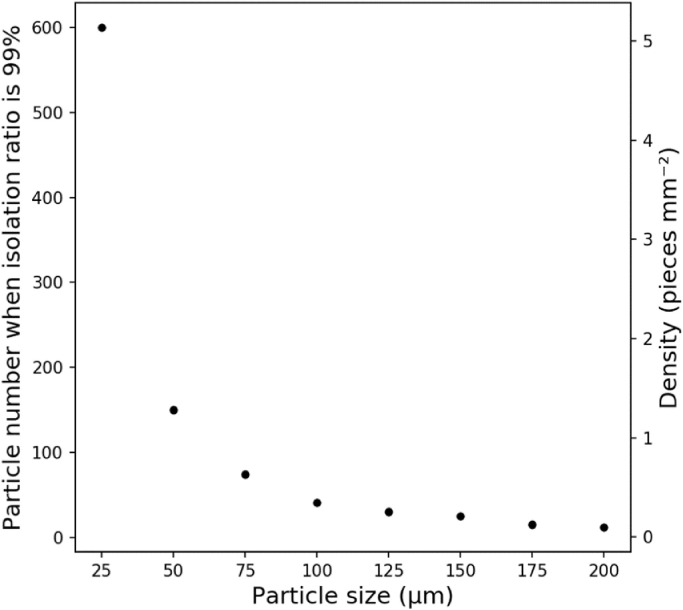
Particle number when the isolation ratio was 99% for each particle size.

When making a microscopy measurement, overlapping particles on the filter will cause errors in the sizes and number of particles found. We assumed that correct measurements were possible at isolation ratios of ≥99%, meaning the correct number of particles of each particle size would be measured (Fig. 5). The number of particles giving an isolation ratio of 99% was 600 when the particle size was 25 µm but decreased sharply as the particle size increased and was 41 when the particle size was 100 µm. The particle density results are shown in Fig. 5. The particle density at which the isolation ratio was 99% was 5.13 mm^−2^ when the particle size was 25 µm but decreased sharply as the particle size increased and was 0.351 mm^−2^ when the particle size was 100 µm.

The numerical experiments indicated the appropriate number of particles on a filter to allow effective microscopic analysis to be performed. The isolation ratio decreased linearly as the number of particles increased (Fig. 4). The isolation ratio decreased more dramatically as the particle size increased; therefore, the isolation ratio needs to be calculated for a real sample with a high proportion of large particles. Particles with diameters ≥200 µm tended to overlap. Samples containing such particles were considered unsuitable for analysis by microscopy.

An isolation ratio of 99% was assumed to be required for a circular filter with a diameter of 12.2 mm to preclude erroneous measurements. For filters with different diameters, the conditions required to preclude erroneous measurement could be determined by taking the particle density into consideration (Fig. 5). The relationship between the particle size *S* and particle density *D* when the isolation ratio is 99% can be described using the equation:

(2) $$D=4.13e^{-0.02S}\left(r^{2}=0.862,\;\;Pearson\;test;p<0.05\right),$$

where *D* (in particles per mm^2^) is the particle density when the isolation ratio is 99% and *S* is the particle size (µm).

This equation helps us to determine the appropriate number of particles for effective microscopy measurements to be made for filters of various sizes.

**Fig. 6:**
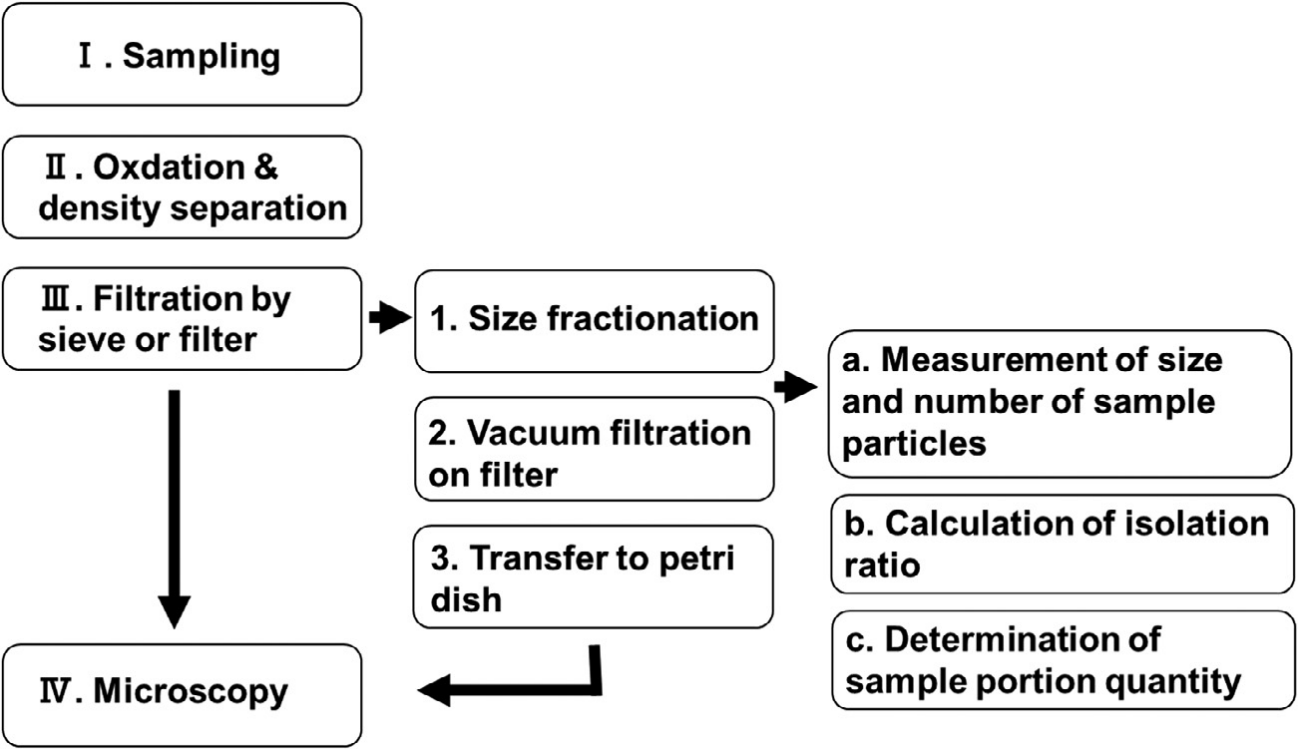
General procedure for analyzing small microplastics including the process for determining the appropriate amount of sample to analyze. The new steps between size fractionation and vacuum filtration are labeled a–c. Using steps a–c make it possible to make correct measurements.

The isolation ratios for various numbers and particle sizes can be estimated by performing numerical experiments. When analyzing a real sample, we propose that the procedure described below be performed prior to filtration of the sample that has been subjected to the oxidation treatment and density separation process (Fig. 6). First, a small subsample should be taken and (a) the particle concentration and particle size distribution measured; then (b) the isolation ratio for the filter should be determined by performing numerical experiments; and finally (c) the appropriate portion of the sample to pass through the filter for effective microscopy analysis should be determined.

Finally, we attempted an analysis using a field sample. This sample was collected from one point in Tokyo Bay (12 Dec 2020, 35° 30.72′ N, 139° 49.89′ E). The collected sample was subjected to oxidation treatment and density separation, and the particle size distribution was examined. The particle size distribution of the Tokyo Bay sample is shown in Fig. S1 (Supplementary materials), and the average particle size was 93.4 µm. The ratio of the particle number concentrations of 60 µm, 80 µm, 100 µm, 120 µm, and > 130 µm in the particle size distribution was 7:2.95:3.05:1:1. Numerical experiments were conducted by appropriately applying the ratio of this particle size distribution to the number of particles. At this time, 200 µm was set as the particle size of > 130 µm. An appropriate number of particles with an isolation ratio of 99% was determined. Eventually, we determined that the appropriate number of particles on a filter with a diameter of 12.2 mm is 60 in total (i.e., a ratio 60–80–100–120 µm to > 130 is 27:11:13:4:5 particles).

This will make the measurements of the number of particles and particle sizes by microscopy more reliable. Furthermore, when filtering the sample, it should be kept in mind that the sample distribution on the filter will not be biased.

## Declaration of Competing Interest

The authors declare that they have no known competing financial interests or personal relationships that could have appeared to influence the work reported in this paper.

## Appendix Supplementary materials

Image, application 1
