# Supplementary material for: Determining the appropriate number of particles on a filter to allow small microplastics to be analyzed by microscopy
Source: MethodsX. 2022 Feb 24;9:101646. doi: 10.1016/j.mex.2022.101646 (PMC8914551; doi:10.1016/j.mex.2022.101646)
Supplement: Supplementary file 1 [file mmc1.docx]

Supplementary materials:

Program:

The core code consists of three sections, with one function each. The first “randomParticle(self)” function can realize the purpose of generating random x and y coordinates, which also means that the points/coordinates will be uniformly distributed without center-preference or edge-preference in the circle created in the second function. The second “filterCircle” function is used to create the circles in the background filter. The circles are determined by the x and y coordinates and particle diameter. The last “NotOverlapping” function is the most important one and is used to calculate the not-overlap rate, which is directly correlated to the appropriate number of particles. The algorithm only calculates how many are particles not overlapping, without counting the overlapping particles, because the overlap situation may vary (double overlap, triple overlap, quadruple overlap, etc.), complicating the algorithm.

Part 1./////////////////////////////////////////////////////////////////////////////////////////////////////////////////////////////

def randomParticle(self):

n1=np.random.random(size=self.particleNumber)

n2=np.random.random(size=self.particleNumber) # n2 can be equel to to n1

theta=2*np.pi*n1

rr=(DiameterFilter-self.particleDiameter)/2*np.sqrt(n2)

#Randomly Generated, Uniformly Distributed

x=rr*np.cos(theta)

y=rr*np.sin(theta)

return x,y,self.particleDiameter

def filterCircle(LIST,pixels):

# Create the Background

plt.figure(figsize=(10,10),dpi=pixels)

# 10 units, 72 pixels / unit, 72 is the default number

angle=np.linspace(0,2*np.pi,1500) plt.plot(DiameterFilter/2*np.cos(angle),DiameterFilter/2*np.sin(angle),color='black')

#12.2 is the diameter of lab's funnel

plt.xlim=(-6.5,6.5)

plt.xticks([-8,-4,0,4,8])

plt.ylim=(-6.5,6.5)

plt.yticks([-8,-4,0,4,8])

plt.xlabel('x (mm)')

plt.ylabel('y (mm)')

plt.title('Simulating Particle Distribution on Filter')

for i in np.arange(len(LIST)):

for j in range(len(LIST[i][0])):

c=plt.Circle(xy=(LIST[i][0][j],LIST[i][1][j]),radius=LIST[i][2]/2)

plt.gca().add_artist(c)

plt.grid(True)

plt.gca().set_aspect('equal',adjustable='box')

plt.show()

def NotOverlapping(List,LIst):

#List is a 2D array, LIst is a 3D array

# If Overlaps, Number Plus 1. Finally, Check the total sum of number.

# Tangent situation is overlap

# Only know the not-overlapping rate without knowing overlapping situation

num1=0

num2=0

num3=0

num4=0

num5=0

Len=len(List[0])

length=[0]

Length=0

for g in range(5):

a=len(LIst[g][0])

Length+=a

length.append(Length)

for i in range(length[0],length[1]): #check the particles of first condition

Loop=0

for j in range(length[0],length[1]): #comparing with the rest of this size

if i != j:

if (List[0][i]-List[0][j])**2 + (List[1][i]-List[1][j])**2 <= ((List[2][0]+List[2][0])/2)**2:

break

else:

Loop+=1

else:

continue

for h in range(1,5):

#comparing with other 4 different sizes

for k in range(length[h],length[h+1]):

if (List[0][i]-List[0][k])**2 + (List[1][i]-List[1][k])**2 <= ((List[2][0]+List[2][h])/2)**2:

break

else:

Loop+=1

else:

continue

if Loop==Len-1:

num1+=1

//////////////////////////////////////////////////////////////////////////////////////////////////////////////////////////////

Particle size distribution of field survey result:


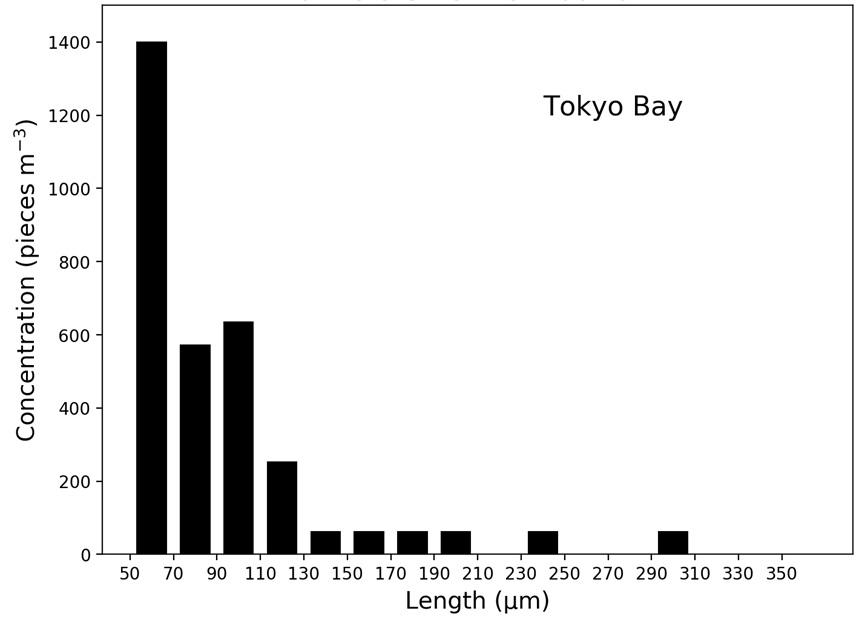


Fig. S1. Particle size distribution of microplastic particles less than 350 µm in Tokyo Bay.
